# Higher Hamstrings Strength and Stability Are Related to Lower Kinematics Alteration during Running after Central and Peripheral Fatigue

**DOI:** 10.3390/s22051990

**Published:** 2022-03-03

**Authors:** Alberto Encarnación-Martínez, Antonio García-Gallart, Roberto Sanchis-Sanchis, Irene Jimenez-Perez, Jose I. Priego-Quesada, Pedro Pérez-Soriano

**Affiliations:** 1 Research Group in Sports Biomechanics (GIBD), Department of Physical Education and Sports, University of Valencia, 46010 Valencia, Spain; roberto.sanchis@uv.es (R.S.-S.); i.jimenez.gibd@gmail.com (I.J.-P.); j.ignacio.priego@uv.es (J.I.P.-Q.); pedro.perez-soriano@uv.es (P.P.-S.); 2 The Civil Guard, Secretary of State for Security, Ministry of the Interior, 28010 Madrid, Spain; garciagallart@gmail.com

**Keywords:** running, fatigue, strength, stability, kinematics

## Abstract

Fatigue can be classified as peripheral or central depending on the extent of its effects. Muscle strength reduction, associated with the appearance of fatigue during running, produces kinetics and kinematics modifications which could lead to an increased risk of injury. This study aimed to analyze the effect of peripheral and central fatigue protocols in running kinematics and to investigate the relationship between isokinetic strength and dynamic stability in fatigue related changes. Eighteen male recreational runners participated in the study. The dynamic postural stability index (DPSI) and quadriceps and hamstring isokinetic strength were assessed before the fatigue test. Then, angular kinematics during treadmill running were evaluated in pre- and post-fatigue states (central and peripheral). The results showed that runners with higher hamstring isokinetic strength and better DPSI had lower modifications after central fatigue of stance time, knee flexion, vertical and leg stiffness, and ankle dorsiflexion during the absorption and propulsion phases (r > 0.400, *p* < 0.05). Moreover, small changes in ankle dorsiflexion at initial contact after peripheral fatigue are related to a better DPSI and higher hamstring isokinetic strength (r > 0.400, *p* < 0.05). In summary, high values of hamstring isokinetic concentric strength and dynamic stability are related to lower increases of range of movements during running after central and peripheral fatigue. So, fatigue may affect to a lesser extent the running technique of those runners with higher hamstring strength and stability values.

## 1. Introduction

Neuromuscular fatigue has been suggested as one of the main causes of injury in running [1] since it is characterized by decreasing muscle strength or power and produces kinetics and kinematics modifications [1,2,3,4,5,6,7]. Neuromuscular fatigue is commonly classified as peripheral and central to clarify the origin of these changes. Peripheral fatigue causes alterations at a muscular level and in contractile elements (e.g., alteration at the cross-bridges level, sarcolemma excitability, or excitation–contraction coupling failure) [8,9]. On the other hand, central fatigue is produced by limitations at the neuromuscular junction (e.g., limiting maximal voluntary activation or neural drive to the muscle) [8,9].

Peripheral fatigue during running decreases muscle strength and activity [3], modifies running biomechanics and spatiotemporal parameters [3,10], and increases ground reaction forces [10] and shock absorption [3].

Central fatigue, also alters muscle strength and activity [11,12,13], promoting changes in movement patterns and spatiotemporal parameters [11,14], increases in ground reaction forces [15] and shock absorption [16], as well as decreases in stiffness characteristics [17], increases in tissue vibration [18] and plantar pressures [19], and decreased postural stability dynamics [20], trunk stability [21,22] or in the performance of cognitive tasks [23].

In addition to fatigue, some of these changes are also affected by the surface [24,25], footwear [26], orthoses [27] or compression garments [16].

Excessive vertical ground reaction forces (vGRF) maintained for a long time during running causes important stress in the musculoskeletal system, and its capacity to sustain these forces can be reduced because of the fatigue [1,3,4,5]. For this reason, vGRF is considered a relevant outcome parameter for running assessment since high peak values or a loading rate that is higher than the runner capacity increases injury risk [28,29]. In order to minimize vGRF after both peripheral [3,4] and central fatigue [1,5], an increase of range of movements is produced as a compensatory strategy, characterized by a greater knee-flexion angle during ground contact.

It has been suggested that hamstrings muscles play an essential role during running fatigue and injury risk because an inhibition of the hamstring muscles is produced before the onset of fatigue, which causes a dominance of the quadriceps in the loading response phase and that induces an increased knee flexion [3,4]. However, these modifications can increase the metabolic cost, making the running technique less efficient [5,6]. So, high levels of muscle strength could prevent, or at least delay, the kinematic changes associated with fatigued running [30]. 

During forward jump landing tasks, dynamic postural control imitates the initial contact and absorption phases of running, where knee flexion–extension strength has a leading role in a safe landing [31]. It has been shown that fatigue also increases the range of movements and decreases vGRF in this type of task [32,33]. 

Biomechanics changes due to fatigue lead to suboptimal movements that can increase the risk of injury [19]. In addition, injuries occur especially nearly at the end of competitions or training where the fatigue processes are very advanced [34].

Therefore, the high popularity of running and the high injury incidence suggest that identifying and comparing the biomechanical changes produced by peripheral and central fatigue, depending on factors such as strength or stability, could add a further step in the prevention of running injuries.

We hypothesized that after fatigue protocols the running kinematics will change, adopting a less efficient running pattern. We also expected that central fatigue will affect the running kinematics more than peripheral fatigue protocol, showing greater changes during landing and absorption phases during central fatigue. While we expect to observe compensatory patterns to maintain running efficiency after peripheral fatigue. Different levels of strength and/or dynamic stability would be hypothesized to affect running kinematics after fatigue, specifically runners with a higher isokinetic strength and/or higher dynamic stability would reduce the kinematic changes expected after central and/or peripheral fatigue protocols. Describing the relationship between the appearance of fatigue (central and peripheral), the alterations that it produces on the running kinematics, and the levels of strength and stability, would be an advance in the understanding of the internal processes related to the factors of running injury risks.

We explored the running kinematics before and after two fatigue protocols (central and peripheral) and related to leg flexion–extension the isokinetic strength profile and dynamic stability. Our objective was to quantify running kinematic changes because of central and peripheral fatigue protocols and to relate the magnitude of the changes to the levels of isokinetic strength and dynamic stability. The key contributions of this paper can be summarized as follows:

- We investigated the differences in running kinematics after two fatigue protocols to identify the responses associated with fatigue.
- We have described that central fatigue induce changes in running kinematics to a lesser efficiency running pattern.
- We described the relationship between isokinetic strength and dynamic stability variables as a predictor of prevention effects of the fatigue processes.
- We have described that higher hamstring isokinetic strength and dynamic stability are related to lower kinematic changes in the running pattern.

## 2. Materials and Methods

### 2.1. Participants

Eighteen male recreational runners (*n* = 18) participated in the study (age: 28.2 ± 8.6 years; height: 1.77 ± 0.065 m; body mass: 71.7 ± 8.4 kg; estimated maximal oxygen consumption: 62.2 ± 4.7 mL/kg/min; running experience: 7.3 ± 5.3 years). 

For the selection of the sample, a non-probabilistic sampling by quotas was used, whose quotas were represented by the inclusion and exclusion criteria. As inclusion criteria for the study, it was decided that all the participants were men, recreational runners, aged between 18 and 45 years, who ran regularly [35], at least twice a week in the last year [36], and who did not present lesions at the time of the investigation or in the 6 months prior to it [35]. Participants who did not meet all the criteria set were excluded. The inclusion of the athletes who met the conditions to participate in the study was carried out once the informed consent to participate in the project had been granted. Informed consent was provided to all participants before inclusion in the study, which was approved by the Ethics Committee of the University (registry number: 6775). A sample size calculation was performed based on the ANOVA repeated measures within factors design, using the G-Power 3 software (version 3.1.9.7, Düsseldorf, Germany). This analysis indicated that at least a sample of 16 cyclists was required to detect significant differences in the different variables analyzed with a minimum detectable effect size of f = 1.0 (large) (α = 0.05, β = 0.05, power = 0.94).

### 2.2. Experimental Setups

**Figure 1:**
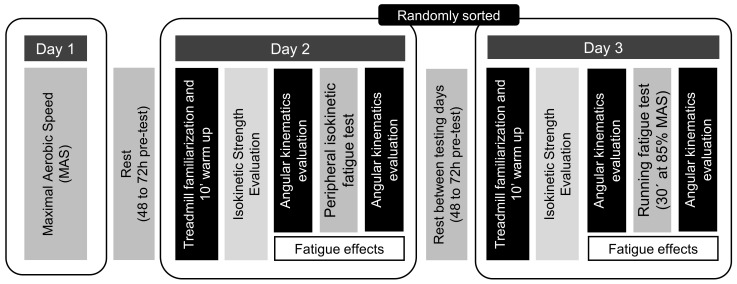
Experimental protocol followed in the study.

Each participant completed three evaluation sessions, separated by 48–72 h each one. In the first session, the maximal aerobic speed (MAS) was estimated using the 5-min Running Field Test [37]. The second session was carried out as follows: (I) warm-up, (II) isokinetic strength registration, (III) angular kinematics recording before and after peripheral fatigue. Finally, the third session was performed as follows: (I) warm-up, (II) evaluation of dynamic postural stability, (III) angular kinematics recording before and after central fatigue. It should be noted that the second and third laboratory sessions were randomized. All measurements were registered in the dominant limb [38]. The warm-up consisted of running freely for 10 min, which also allowed them to familiarize themselves with the treadmill (Excite^®^ + Run MD Inclusive, Technogym Trading S.A., Barcelona, Spain) [37] (Figure 1). 

#### 2.2.1. Isokinetic Strength Assessment

Regarding isokinetic strength registration, peak concentric torque values in quadriceps and hamstring muscles were recorded using an isokinetic dynamometer (Biodex System Pro 3™, Biodex Medical Systems, Inc., New York, NY, USA). From a seated position (80° hip flexion), two sets of concentric/concentric knee flexion–extension movements were performed, with a range of motion ranging from 0° (full extension) to 90° of knee flexion [3,39]. In the first set, three sub-maximal concentric contractions at 60°/s were performed as a familiarization. In the second set, three maximal concentric contractions at 120°/s were carried out to determine peak concentric torque values. Peak concentric torque values in quadriceps (QTORQ) and hamstrings (HTORQ) were registered, considering the highest value for analysis, and expressed as a percentage of the body weight. The angles at which QTORQ and HTORQ were reached were also recorded (QANG-TORQ and HANG-TORQ, respectively). The hamstrings/quadriceps strength ratio (H/Q ratio) was also calculated.

#### 2.2.2. Dynamic Stability Assessment

Dynamic postural stability was registered through an adaptation of the Dynamic Postural Stability Index (DPSI) test [40]. Before the test, each participant performed three valid countermovement jumps to calculate the 50% of their maximum jump height, using the highest jump [40]. Runners were placed 70 cm from the center of a force platform (Kitsler 9286BA, Kistler Group, Winterthur, Switzerland) and they were instructed to double limb jump over an elastic band set at 50% of their maximum jump height, with hands on hips and looking forward, landing on their dominant limb, and stabilizing as quickly as possible. To familiarize themselves with the test, a minimum of three practice attempts were required [41]. After the practice, three attempts were performed to evaluate the mediolateral (MLSI), anteroposterior (APSI), vertical (VSI), and global (DPSI) stability indices [40], recording the ground reaction force (GRF) signals at a frequency of 1000 Hz. The first three seconds after impact were used for analysis [40]. Isokinetic strength and dynamic postural stability descriptive variables are shown in Table 1.

#### 2.2.3. Angular Kinematics Assessment

**Figure 2:**
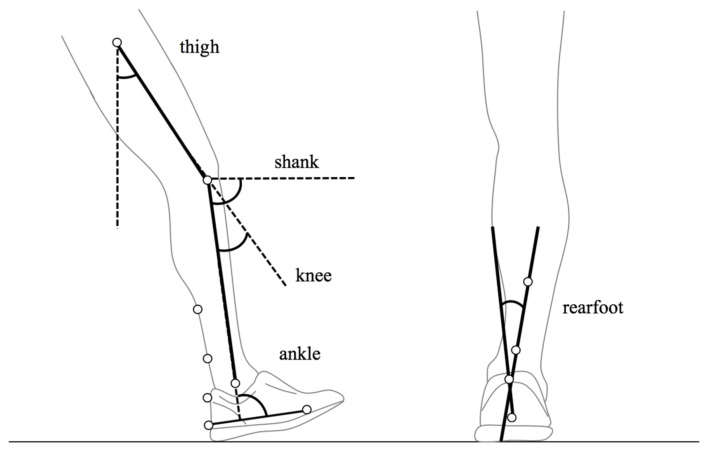
Kinematic markers setup model employed in the study.

Regarding angular kinematics recording, the measurement protocol was the same in both sessions, modifying only fatigue protocol (peripheral or central fatigue). Angular kinematics were recorded in a 2-min treadmill running period at 3.89 m/s and 0% slope, both before and after fatigue conditions. Retro-reflective markers were placed on the lateral of the greater trochanter, femoral condyle, lateral malleolus and 5th metatarsal head. Four posterior markers were also located on the shoe and lower leg [42] (Figure 2). An Optitrack V120:Trio infrared motion capture system (NaturalPoint, Inc., Corvallis, OR, USA), running at 120 Hz, was used during the last 30 s of each 2-min period (pre and post-fatigue) to track the markers. No recovery time was allowed. Once they finished central/peripheral fatigue protocol, the 2-min running test was performed as quickly as possible to avoid recovery processes.

Forty-five stride cycles were approximately registered in each condition, and data processing was performed using Motive software (NaturalPoint, Inc., Corvallis, OR, USA). Marker data were filtered with a fourth-order low-pass Butterworth filter with a cut-off frequency of 6 Hz. A custom routine performed with the MatLab R2013b program (Mathworks Inc., Natick, MA, USA) was used to calculate running kinematics. The angle convention (Figure 2) was used to detect the movements of thigh and knee flexion–extension, shank oscillation, ankle dorsiflexion-plantar flexion and rearfoot eversion–inversion. Standing calibration of body segments was considered as zero degrees. Thus, positive values represented hip flexion, knee flexion, greater shank oscillation, ankle plantar flexion and rearfoot inversion, while negative values described hip extension, knee extension, lower shank oscillation, ankle dorsiflexion and rearfoot eversion. Root mean square error (RMSE) was calculated to determine the 3D reconstruction accuracy, obtaining a systematic error of 0.005, 0.012 and 0.037 mm for X (mediolateral), Y (anteroposterior), and Z (vertical) axes, respectively.

Gait cycles were normalized to 101 data points, and the stance was divided into absorption and generation phases. The absorption phase was represented from the initial contact (IC) to maximum knee flexion (MKF) in the midstance, while the generation/propulsion phase was interpreted from MKF to toe-off (TO) [43]. The best method to identify the IC regardless of the foot strike pattern is through the vertical velocity of the pelvis [44]. So, IC was identified as the frame of maximum downward velocity of the trochanter. MKF was detected as the peak knee flexion located between the two peaks knee extensions produced in IC and TO. TO was identified as the second peak knee extension [44]. Finally, maximum oscillation during swing (MO) as peak knee flexion located between the two peaks knee extensions produced in the TO and IC. Additionally, spatiotemporal parameters (stride frequency, stride length, stride time, stance time, and swing time) were calculated. Finally, leg and vertical stiffness were also estimated from the kinematics variables using the spring-mass model [45].

#### 2.2.4. Fatigue Protocols

Regarding fatigue generation, on the one hand, central fatigue was induced by 30-min of treadmill running (0% slope) at 85% of MAS [37]. Furthermore, runners had to manifest a perceived effort equal to or greater than 17 or “Very Hard” [2] on the Borg’s Scale 6–20 [46]. On the other hand, peripheral fatigue was induced with an isokinetic dynamometer. Continuous concentric/concentric knee flexion–extension movements at 120°/s were performed, exerting maximal effort through the whole range of motion, without rest. Fatigue protocol finished when the concentric peak torque fell below 50% for 3 consecutive movements in both directions [39].

### 2.3. Statistics

Data were analyzed with the statistics software SPSS Statistics (SPSS v.26, Chicago, IL, USA). After checking the normality of the variables with the Kolmogorov–Smirnov test, two-way repeated measures ANOVA (normal distribution variables) or Friedman test (non-normal distribution variables) was carried out to compare running kinematics (I) pre vs. post central fatigue and (II) pre vs. post peripheral fatigue. Delta (Δ) or pre-post fatigue modifications between peripheral and central fatigue were evaluated by paired samples *t*-test (normal distribution variables) or Wilcoxon test (non-normal distribution variables). Statistical significance was set at *p* < 0.05. When differences were significant, confidence intervals (95% CI) and Cohen’s d effect size (ES) were also calculated, where >0.2 is considered small, >0.5 moderate, and >0.8 large [47].

The relationship between research factors and post-fatigue variables was evaluated through Pearson’s Correlation Coefficient (r), where magnitude was interpreted as: <0.1, trivial; 0.1–0.3, small; 0.3–0.5, moderate; 0.5–0.7, large; 0.7–0.9, very large; 0.9–1.0, almost perfect; and 1.0, perfect [48]. Moreover, the coefficient of determination (R^2^) (i.e., the percentage of the variance in the dependent variable that can be explained by variations in independent variables) was calculated elevating r squared and multiplying it by 100 [49].

## 3. Results

Kinematics modification pre vs. post peripheral and central fatigue, and the comparison of kinematics modifications after central vs. peripheral fatigue, are shown in Table 2 and Table 3, respectively. Regarding the effects of fatigue on spatiotemporal variables, in our study, only stance time and propulsion time was significantly higher after central fatigue (*p* = 0.025 and *p* = 0.033, respectively) (Table 2). No differences were observed on stiffness variables.

**Figure 3:**
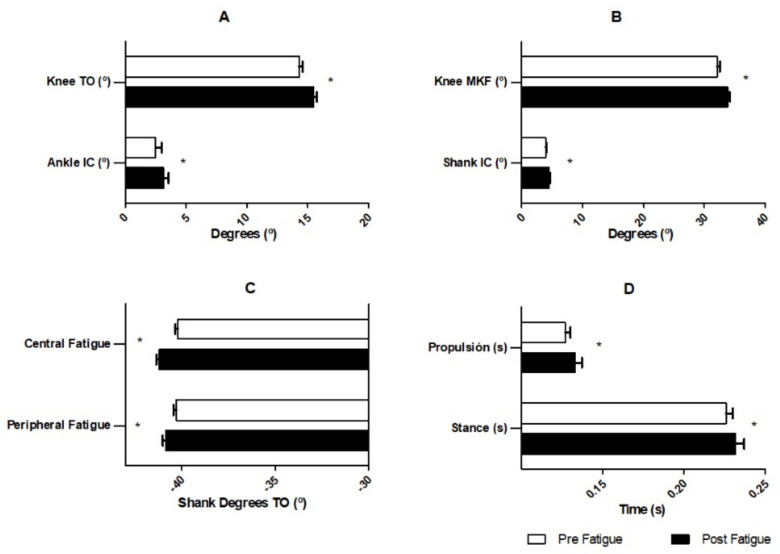
Statistical differences between pre and post fatigue protocols. (**A**): Kinematics differences after peripheral fatigue protocol; (**B**): kinematic differences after central fatigue protocol; (**C**): shank differences after central and peripheral fatigue protocols; (**D**): spatiotemporal differences after the central fatigue protocol. *: Statistically differences (*p* < 0.05) between pre and post fatigue.

At initial contact, shank inclination (*p* = 0.034) and ankle-flexion (*p* = 0.035) were increased after central fatigue and peripheral fatigue, respectively. Knee-flexion was increased (*p* = 0.000) after the central fatigue protocol during the maximum knee flexion phase. During the take-off phase, knee-flexion increased (*p* = 0.003) after the peripheral fatigue protocol, as well as the shank inclination (*p* = 0.020), which also increased after the central fatigue protocols (*p* = 0.002) (Figure 3). No differences were observed on maximum oscillation during the swing phase (Table 2).

**Figure 4:**
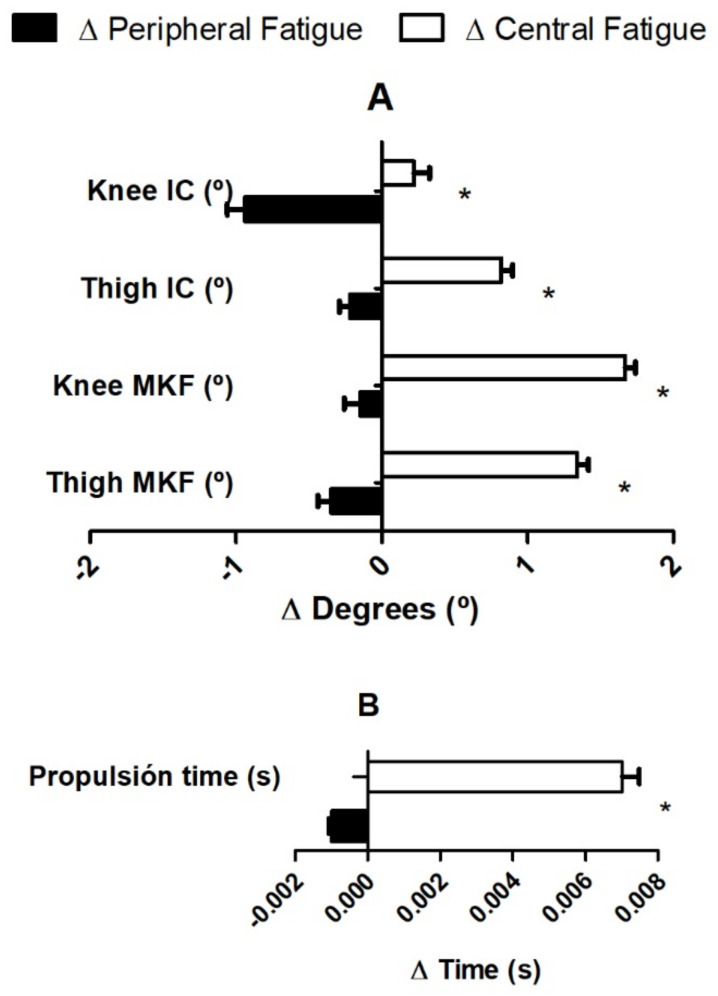
Change results between central and peripheral protocols. (**A**): Angular kinematics difference changes after fatigue protocols; (**B**): spatiotemporal difference changes after fatigue protocols. Black bars: changes due to peripheral fatigue; white bars: changes due to central fatigue. *: Statistically differences (*p* < 0.05) between pre and post fatigue.

The comparison between the changes produced by both types of fatigue (Δ central vs. Δ peripheral) showed that the propulsion time was higher after central fatigue (*p* = 0.001) (Figure 4B). Likewise, the increases in joint range of motion (ROM) produced by central fatigue were greater, compared to peripheral fatigue (Figure 4A), in the hip (*p* = 0.027 for IC, *p* = 0.008 for MKF) and knee joint (*p* = 0.047 for IC, *p* = 0.009 for MKF) in the phases of initial contact and maximum knee flexion, respectively (Figure 4 and Table 3).

Regarding the relationship between kinematics modification after both fatigues and muscular strength and stability, the Pearson’s Correlation Coefficient showed that modifications due to central fatigue (pre vs. post) in the knee flexion during MKF were positively related to higher DPSI values (poor performance) (r = 0.534; *p* < 0.001, R^2^ = 28.5) and negatively with HTORQ (r = −0.582; *p* < 0.001, R^2^ = 33.9). In addition, stance time was positively related to higher DPSI values (poor performance) (r = 0.479; *p* = 0.002, R^2^ = 22.9) and negatively with HTORQ (r = −0.474; *p* = 0.002, R^2^ = 22.5).

In the peripheral fatigue, the ankle dorsiflexion during the IC was positively related to HROM (r = 0.480; *p* = 0.002, R^2^ = 23.0) and HTORQ (r = 0.603; *p* < 0.001, R^2^ = 36.4), and negatively to higher DPSI values (poor performance) (r = −0.469; *p* = 0.004, R^2^ = 22.0). Moreover, in the TO, the knee flexion was negatively related to HTORQ (r = −0.647; *p* < 0.001, R^2^ = 41.9).

If the values after central fatigue and after peripheral fatigue are compared, the ankle dorsiflexion in the MKF was negatively correlated with higher DPSI values (poor performance) (r = −0.497; *p* = 0.001, R^2^ = 24.7) and positively to HTORQ (r = 0.409; *p* = 0.010, R^2^ = 16.7). Furthermore, leg and vertical stiffness were negatively correlated with higher DPSI values (poor performance) (kLeg: r = −0.435; *p* = 0.006, R^2^ = 18.9; kVert: r = −0.455; *p* = 0.004, R^2^ = 20.7) and positively to HTORQ (kLeg: r = 0.460; *p* = 0.003, R^2^ = 21.2; kVert: r = 0.451; *p* = 0.004, R^2^ = 20.3).

In delta modifications, HTORQ were negatively correlated to delta thigh flexion in the IC (r = −0.565; *p* = 0.023, R^2^ = 31.9) and higher DPSI values (poor performance) were positively correlated to delta propulsion time (r = 0.566; *p* = 0.022, R^2^ = 32.0, respectively).

## 4. Discussion

The aim of this study was to analyze the relationship between isokinetic strength and dynamic postural stability with the pattern of movements during running after peripheral and central fatigue. The main results were that runners with higher hamstring isokinetic strength and a better DPSI had lower modifications after central fatigue of stance time, knee flexion, vertical and leg stiffness, and ankle dorsiflexion during the absorption and propulsion phases. Moreover, small changes in ankle dorsiflexion at initial contact after peripheral fatigue are related to a better DPSI and higher hamstring isokinetic strength.

The body’s ability to sustain the GRF received from the ground could decrease as fatigue increases, as a necessary corporal compensatory strategy, such as knee flexion, increases to reduce the GRFs [1,5,7]. When hamstring muscles are fatigued, the quadriceps/hamstring muscular activation ratio is modified in favor of the quadriceps, increasing knee-flexion angles to achieve a greater quadriceps muscle capacity to generate strength and absorb impacts [3,4]. This compensatory response to fatigue could reduce 68 N of vGRFs, approximately, for each knee angle increased [7]. In our study, a mean difference of 1.67 degrees in knee flexion (95% CI: 0.5 to 3.1°) was registered after the central fatigue protocol, which would mean an estimated reduction of the GRF of approximately 113.6 N (95% CI: 34 to 210.8 N).

The increase of ranges of movement affects the knee joint and could be accompanied by an increase in hip flexion and ankle dorsiflexion during the phase of maximum absorption [2]. It would aim to maintain balance and reduce received forces [1,7], diminishing knee loading without increasing energetics or biomechanics demand in ankle [50]. These kinematics modifications in a fatigue state, derived from hamstring fatigue [3,4] (among others), could coincide with our results since there was a relationship between lower hamstrings strength values and greater increases of the range of movements during the absorption phase after central fatigue. Moreover, the increased range of movement of thigh flexion, knee flexion and ankle dorsiflexion during the absorption phase showed in the central fatigue state could increase the stance time [51]. It would explain the reduction of leg and vertical stiffness after central than peripheral fatigue due to the leg compression increase [52]. However, although these compensatory strategies decrease the vGRF, it can also increase the metabolic cost [5], since it has been shown that VO^2^ could increase by 25% for each 5° increase in the midstance knee flexion angle [6].

Hayes, Bowen and Davies [30] concluded that local muscular endurance of concentric hip extensors and eccentric knee flexors (principal actions of hamstring muscles) are important in maintaining a stable running style (stride mechanics), preventing or delaying the kinematic changes associated with fatigued running. In the same way, Kellis, Zafeiridis and Amiridis [3] affirmed that kinematics modifications during fatigued running are the result of muscle-performance impairments and contribute to the runner’s inability to maintain the same technique for a long period of time. Our results could agree with this line of knowledge because we found that higher hamstring strength values are related to lower kinematics modifications in the fatigue condition, or angular positions closer to the pre-fatigue condition.

The relationship between the DPSI test and angular kinematic and leg compression modifications after central fatigue was similar to the relationship between it and the hamstring isokinetic strength. These forward jump landing tasks simulate the initial contact and absorption phases of running. Moreover, it has been shown that fatigue also caused a change in landing strategy, increasing the range of movements to decrease vGRF in the IC after landing [32,33], similar to what happens during fatigued running. Wikstrom, Powers and Tillman [31] described the leading role of hip and knee muscle strength and lower extremity neuromuscular control in the kinetic energy decrease, the body’s downward velocity to zero, and performing a safety landing. Further, Williams et al. [41] also emphasized the importance of thigh musculature, suggesting that deficits in knee flexion–extension strength could decrease dynamic postural stability and increase the risk of injury. So, our results may support this theory because we found a relationship between better dynamic stability and running kinematic closer to the pre-fatigue values or minor increases of range of movements in the central fatigue state.

After peripheral fatigue, runners adopted a hip and knee position more extended during the initial contact and maximum absorption than in the pre-fatigue condition (non-significant differences), and also compared after peripheral vs. central fatigue (significant differences). Besides, the ankle plantarflexion was increased in the IC. Gerritsen, van den Bogert and Nigg [7] showed that GRFs could be reduced by 85 N, approximately, for each plantarflexion angle increased. Therefore, localized quadriceps and hamstring fatigue might have forced the body system to adopt a different strategy from that used in the central fatigue state in order to reduce GRF, showing greater plantarflexion. According to our results, these modifications might be lower with better stability after landing.

Regarding the propulsion phase, central fatigue increased the time in this phase significantly more than peripheral fatigue. Gastrocnemius muscles play an important role in maintaining force production during the propulsion phase when thigh muscles are fatigued [53]. Paavolainen et al. [54] showed that the capacity of the neuromuscular system to store and use the elastic energy in the fatigue state was higher in well-trained runners. This could coincide with our results since a better DPSI performance was related to lower increases in the propulsion time.

In peripheral fatigue, the posterior shank oscillation increased during TO. The lower force production capacity of hamstrings to extend the hip and flex the knee is one of those responsible for the fatigue modifications in the toe-off [4]. So, the relationship between higher hamstring isokinetic strength values and greater shank posterior oscillations could explain the maintenance or lower decrease of force production necessary to sustain the speed.

The study is not without limitations. There are studies that analyze the effects of fatigue on 3D kinematics during running using a sample size less than or equal to that used in the present investigation [1,2,30]. Moreover, a large effect size was observed in most of the differences assessed, suggesting that the sample size was enough for this study [55]. However, a higher sample size could describe more precisely the relationship between hamstrings strength and stability and kinematics modifications after peripheral and central fatigue. Therefore, we think it would be interesting to carry out more research in the future with a larger number of runners. This would allow us to separate them into different groups according to their strength and stability levels, and analyze the kinematic changes in the fatigue state and the energy cost by a gas analyzer. Moreover, it would also be interesting to investigate whether a training program aimed at increasing hamstring strength and stability could reduce or delay the fatigue effects on increasing ranges of movement and metabolic cost.

## 5. Conclusions

In conclusion, fatigue protocols which induced running kinematics changes were either peripheral or central fatigue. Greater changes were observed after central fatigue, making the running pattern less efficient as the knee become more flexed after the fatigue protocol.

Runners with higher hamstrings isokinetic concentric strength and a better DPSI had lower increases of ROM during the absorption phase and propulsion time after central fatigue, as we hypothesized.

After peripheral fatigue, runners with higher hamstring isokinetic concentric strength and a better Dynamic Postural Stability Index showed less ankle dorsiflexion in the initial contact and shank posterior oscillation during the toe-off, respectively.

Therefore, as a practical application, hamstring isokinetic strength and dynamic stability are related to lower kinematic changes in the running pattern after fatigue (central and peripheral), so strength and stability training could prevent or delay the kinematic modifications associated with fatigued running and prevent the early increase in metabolic cost.

## Figures and Tables

**Table 1:** Descriptive parameters of isokinetic strength and Dynamic postural stability.

	Mean	SD
VSI *	0.325	0.056
MLSI *	0.114	0.010
APSI *	0.031	0.005
DPSI *	0.346	0.055
QTORQ (%)	245.28	39.60
HTORQ (%)	124.77	31.26
QANG-TORQ (°)	56.71	5.08
HANG-TORQ (°)	40.65	11.28
H/Q ratio (%)	50.6	8.2

SD: Standard Deviation, *: Dimensionless, VSI: Vertical Stability Index, MLSI: Mediolateral Stability Index, APSI: Anteroposterior Stability Index, DPSI: Dynamic Postural Stability Index, Q: quadriceps, H: hamstrings, TORQ: Peak Torque, ANG: Peak Torque Angle.

**Table 2:** Kinematics modification pre vs. post peripheral and central fatigue.

	Peripheral Fatigue				Central Fatigue			
	Pre-Fatigue	Post-Fatigue	95% CI	Effect Size	Pre-Fatigue	Post-Fatigue	95% CI	Effect Size
	Mean ± SD	Mean ± SD		(Cohen’s D)	Mean ± SD	Mean ± SD		(Cohen´s D)
Stride Freq. (Hz)	177.18 ± 2.48	176.65 ± 2.47			176.39 ± 2.34	174.89 ± 2.14		
Stride Length (m)	2.63 ± 0.15	2.63 ± 0.15			2.65 ± 0.14	2.68 ± 0.13		
Stride Time (s)	0.679 ± 0.009	0.681 ± 0.009			0.682 ± 0.009	0.688 ± 0.009		
Stance Time (s) ^§^	0.222 ± 0.005	0.223 ± 0.005			0.226 ± 0.004	0.232 ± 0.005 *	−0.009/−0.003	1.325
Swing Time (s)	0.457 ± 0.009	0.459 ± 0.008			0.457 ± 0.008	0.456 ± 0.008		
Stance Time (%)	32.71 ± 0.72	32.67 ± 0.60			33.13 ± 0.54	33.76 ± 0.65		
Swing Time (%)	67.29 ± 0.72	67.33 ± 0.60			66.87 ± 0.54	66.24 ± 0.65		
Absorption Time (s)	0.097 ± 0.002	0.098 ± 0.003			0.099 ± 0.002	0.099 ± 0.003		
Propulsion Time (s)	0.125 ± 0.004	0.124 ± 0.004			0.127 ± 0.003	0.133 ± 0.004 *	−0.001/−0.011	0.588
kLeg (kN·m^−1^)	10.96 ± 0.68	10.85 ± 0.62			10.39 ± 0.49	9.83 ± 0.55		
kVert (kN·m^−1^)	27.89 ± 1.21	27.58 ± 1.19			26.82 ± 0.94	25.83 ± 1.03		
Thigh_ IC (°)	24.36 ± 0.8	24.14 ± 0.75			23.77 ± 0.85	24.59 ± 0.98		
Knee IC (°)	12.65 ± 1.22	11.71 ± 1.24			12.34 ± 1.40	12.56 ± 1.40		
Shank IC (°)	3.27 ± 0.75	3.93 ± 0.66			3.85 ± 0.57	4.45 ± 0.74 *	−1.204/−0.054	0.908
Ankle IC (°) ^§^	2.54 ± 1.96	3.15 ± 1.90 *	−0.698/1.918	0.315	0.48 ± 1.67	0.84 ± 1.51		
Rearfoot IC (°)	−0.71 ± 1.75	−1.52 ± 1.82			−0.50 ± 1.76	−1.92 ± 1.41		
Thigh MKF (°) ^§^	16.43 ± 1.17	16.08 ± 1.12			15.61 ± 1.11	16.95 ± 1.12		
Knee MKF (°)	32.29 ± 1.74	32.14 ± 1.72			32.12 ± 1.73	33.79 ± 1.68 **	−2.302/−1.033	0.979
Shank MKF (°) ^§^	−24.30 ± 0.58	−24.56 ± 0.50			−24.09 ± 0.61	−24.42 ± 0.64		
Ankle MKF (°) ^§^	−11.74 ± 1.84	−11.33 ± 1.92			−13.66 ± 1.21	−14.11 ± 1.07		
Rearfoot MKF (°) ^§^	−9.92 ± 2.90	−10.46 ± 4.01			−11.76 ± 1.78	−13.84 ± 1.44		
Thigh TO (°)	−17.50 ± 0.92	−16.86 ± 0.86			−18.30 ± 0.83	−18.48 ± 0.89		
Knee TO (°)	14.33 ± 1.10	15.48 ± 1.12 **	−2.415/−0.602	1.036	14.32 ± 1.20	15.12 ± 1.27		
Shank TO (°) ^§^	−40.27 ± 0.70	−40.84 ± 0.65 *	0.105/1.035	−0.844	−40.19 ± 0.62	−41.18 ± 0.70 **	0.536/1.444	−1.497
Ankle TO (°)	19.06 ± 1.53	19.56 ± 1.48			17.91 ± 1.53	19.20 ± 1.67		
Rearfoot TO (°) ^§^	8.20 ± 3.26	6.14 ± 5.31			6.48 ± 2.93	4.68 ± 3.35		
Thigh MO (°)	18.76 ± 0.92	19.06 ± 0.57			18.00 ± 0.89	18.87 ± 0.81		
Knee MO (°)	92.95 ± 2.80	92.26 ± 2.28			92.29 ± 2.58	93.27 ± 2.41		
Shank MO (°) ^§^	−51.05 ± 12.27	−57.68 ± 12.57			−56.31 ± 13.36	−55.61 ± 13.42		
Ankle MO (°) ^§^	12.75 ± 2.10	13.37 ± 2.17			12.35 ± 2.41	13.00 ± 1.87		
Rearfoot MO (°)	98.2 ± 18.47	103.34 ± 14.92			91.60 ± 20.69	107.19 ± 17.37		

^§^: Variable not adjusted to normality; Friedman test applied; * *p* < 0.05; ** *p* < 0.01; IC: initial contact; MKF: maximum knee flexion; TO: take off; MO: maximum oscillation during swing.

**Table 3:** Comparison of kinematics modifications after central vs. peripheral fatigue.

	Peripheral Post-Fatigue	Central Post-Fatigue	IC 95%	Effect Size	∆ Peripheral Fatigue	∆ Central Fatigue	IC 95%	Effect Size
	Mean ± SD	Mean ± SD		(Cohen’s D)	Mean ± SD	Mean ± SD		(Cohen’s D)
Stride Freq. (Hz)	176.65 ± 2.47	174.89 ± 2.14			−0.526 ± 0.959	−1.503 ± 1.407		
Stride Length (m)	2.63 ± 0.145	2.68 ± 0.133			0.006 ± 0.054	−0.121 ± 0.603		
Stride Time (s)	0.681 ± 0.009	0.688 ± 0.009			0.002 ± 0.004	0.006 ± 0.005		
Stance Time (s) ^§^	0.223 ± 0.005	0.232 ± 0.005 *	−0.012/−0.006	1.800	0.000 ± 0.000	0.010 ± 0.000		
Swing Time (s)	0.459 ± 0.008	0.456 ± 0.008			0.000 ± 0.000	0.000 ± 0.000		
Stance Time (%)	32.67 ± 0.6	33.76 ± 0.65			−0.041 ± 0.28	0.636 ± 0.283		
Swing Time (%)	67.33 ± 0.6	66.24 ± 0.65 *	0.140/2.054	−1.743	0.041 ± 0.28	−0.636 ± 0.283		
Absorption Time (s)	0.098 ± 0.003	0.099 ± 0.003			0.001 ± 0.001	0.000 ± 0.002		
Propulsion Time (s)	0.124 ± 0.004	0.133 ± 0.004			−0.001 ± 0.001	0.007 ± 0.002 **	−0.012/−0.003	5.06
kLeg (kN·m^−1^)	10.85 ± 0.62	9.83 ± 0.55 *	0.812/1.971	−0.438	−0.14 ± 0.21	−0.56 ± 1.04		
kVert (kN·m^−1^)	27.58 ± 1.19	25.83 ± 1.03 *	0.291/3.192	−0.391	−0.38 ± 0.39	−0.98 ± 1.91		
Thigh IC (°)	24.14 ± 0.75	24.59 ± 0.98			−0.22 ± 0.30	0.82 ± 0.32 *	0.424/−1.944	3.353
Knee IC (°)	11.71 ± 1.24	12.56 ± 1.40			−0.94 ± 0.51	0.22 ± 0.46 *	−2.302/−0.015	2.389
Shank IC (°)	3.93 ± 0.66	4.45 ± 0.74			0.66 ± 0.44	0.60 ± 0.36		
Ankle IC (°) ^§^	3.15 ± 1.90	0.84 ± 1.51 *	1.586/3.474	1.346	0.62 ± 0.21	0.36 ± 0.69		
Rearfoot IC (°)	−1.52 ± 1.82	−1.92 ± 1.41			−0.81 ± 1.10	−1.42 ± 1.44		
Thigh MKF (°) ^§^	16.08 ± 1.12	16.95 ± 1.12			−0.35 ± 0.37	1.34 ± 0.33 *	−2.851/−0.520	4.821
Knee MKF (°)	32.14 ± 1.72	33.79 ± 1.68			−0.15 ± 0.46	1.67 ± 0.30 **	−3.105/−0.529	4.687
Shank MKF (°) ^§^	−24.56 ± 0.50	−24.42 ± 0.64			−0.26 ± 0.22	−0.33 ± 0.17		
Ankle MKF (°) ^§^	−11.33 ± 1.92	−14.11 ± 1.07 *	−1.702/3.858	−1.789	0.41 ± 0.29	−0.45 ± 0.56		
Rearfoot MKF (°) ^§^	−10.46 ± 4.01	−13.84 ± 1.44			−0.54 ± 1.67	−2.08 ± 1.46		
Thigh TO (°)	−16.86 ± 0.86	−18.48 ± 0.89			0.64 ± 0.29	−0.18 ± 0.41		
Knee TO (°)	15.48 ± 1.12	15.12 ± 1.27			1.15 ± 0.38	0.80 ± 0.44		
Shank TO (°) ^§^	−40.84 ± 0.65	−41.18 ± 0.70			−0.57 ± 0.27	−0.98 ± 0.19		
Ankle TO (°)	19.56 ± 1.48	19.20 ± 1.67			0.49 ± 0.69	1.29 ± 0.81		
Rearfoot TO (°) ^§^	6.14 ± 5.31	4.68 ± 3.35			−2.06 ± 3.37	−1.80 ± 1.53		
Thigh MO (°)	19.06 ± 0.57	18.87 ± 0.81			0.30 ± 0.59	0.87 ± 0.81		
Knee MO (°)	92.26 ± 2.28	93.27 ± 2.41			−0.69 ± 1.28	0.97 ± 1.18		
Shank MO (°) ^§^	−57.68 ± 12.57	−55.61 ± 13.42			−6.63 ± 7.65	0.70 ± 3.50		
Ankle MO (°) ^§^	13.37 ± 2.17	13.00 ± 1.87			0.62 ± 0.57	0.65 ± 1.29		
Rearfoot MO (°)	103.34 ± 14.92	107.19 ± 17.37			5.14 ± 12.18	15.59 ± 18.55		

^§^: Variable not adjusted to normality; Friedman test applied; * *p* < 0.05; ** *p* < 0.01; IC: initial contact; MKF: maximum knee flexion; TO: take off; MO: maximum oscillation during swing.
